# Retinoic acid and proteotoxic stress induce AML cell death overcoming stromal cell protection

**DOI:** 10.1186/s13046-023-02793-z

**Published:** 2023-08-31

**Authors:** Francesca Liccardo, Martyna Śniegocka, Claudia Tito, Alessia Iaiza, Tiziana Ottone, Mariadomenica Divona, Serena Travaglini, Maurizio Mattei, Rosella Cicconi, Selenia Miglietta, Giuseppe Familiari, Stefania Annarita Nottola, Vincenzo Petrozza, Luca Tamagnone, Maria Teresa Voso, Silvia Masciarelli, Francesco Fazi

**Affiliations:** 1 Department of Anatomical, Histological, Forensic Medicine and Orthopedic Sciences, Section of Histology and Medical Embryology, Sapienza University of Rome, Rome, Italy; 2 Department of Biomedicine and Prevention, University of Tor Vergata, Rome, Italy; 3 Santa Lucia Foundation, I.R.C.C.S., Neuro-Oncohematology, Rome, Italy; 4 Department of Biology, University of Tor Vergata, Rome, Italy; 5 Centro Interdipartimentale-CIMETA, University of Tor Vergata, Rome, Italy; 6 Department of Anatomical, Histological, Forensic Medicine and Orthopedics Sciences, Section of Human Anatomy, Sapienza University of Rome, Rome, Italy; 7 Department of Medico-Surgical Sciences & Biotechnologies, Center for Biophotonics, Sapienza University of Rome, Latina, Italy; 8 Department of Life Sciences and Public Health, Histology and Embryology Unit, Catholic University of the Sacred Hearth, Rome, Italy; 9 Fondazione Policlinico Universitario “A. Gemelli”, IRCCS, Rome, Italy

**Keywords:** AML, Bone marrow stromal cells, Tumor microenvironment, Proteotoxic stress, ER stress, Oxidative stress, YAP, Actin cytoskeleton

## Abstract

**Background:**

Acute myeloid leukemia (AML) patients bearing the ITD mutation in the tyrosine kinase receptor FLT3 (FLT3-ITD) present a poor prognosis and a high risk of relapse. FLT3-ITD is retained in the endoplasmic reticulum (ER) and generates intrinsic proteotoxic stress. We devised a strategy based on proteotoxic stress, generated by the combination of low doses of the differentiating agent retinoic acid (R), the proteasome inhibitor bortezomib (B), and the oxidative stress inducer arsenic trioxide (A).

**Methods:**

We treated FLT3-ITD^+^ AML cells with low doses of the aforementioned drugs, used alone or in combinations and we investigated the induction of ER and oxidative stress. We then performed the same experiments in an in vitro co-culture system of FLT3-ITD^+^ AML cells and bone marrow stromal cells (BMSCs) to assess the protective role of the niche on AML blasts. Eventually, we tested the combination of drugs in an orthotopic murine model of human AML.

**Results:**

The combination RBA exerts strong cytotoxic activity on FLT3-ITD^+^ AML cell lines and primary blasts isolated from patients, due to ER homeostasis imbalance and generation of oxidative stress. AML cells become completely resistant to the combination RBA when treated in co-culture with BMSCs. Nonetheless, we could overcome such protective effects by using high doses of ascorbic acid (Vitamin C) as an adjuvant. Importantly, the combination RBA plus ascorbic acid significantly prolongs the life span of a murine model of human FLT3-ITD^+^ AML without toxic effects. Furthermore, we show for the first time that the cross-talk between AML and BMSCs upon treatment involves disruption of the actin cytoskeleton and the actin cap, increased thickness of the nuclei, and relocalization of the transcriptional co-regulator YAP in the cytosol of the BMSCs.

**Conclusions:**

Our findings strengthen our previous work indicating induction of proteotoxic stress as a possible strategy in FLT3-ITD^+^ AML therapy and open to the possibility of identifying new therapeutic targets in the crosstalk between AML and BMSCs, involving mechanotransduction and YAP signaling.

**Supplementary Information:**

The online version contains supplementary material available at 10.1186/s13046-023-02793-z.

## Background

Acute myeloid leukemia (AML) is a heterogeneous disease caused by a blockage of hematopoietic myeloid precursors differentiation, resulting in the accumulation of immature blasts. The mutational landscape of AML comprises mutations in signaling pathways, transcription factors, epigenetic modifiers, and splicing factors [1]. FMS-like tyrosine kinase 3 (FLT3) internal tandem duplication (FLT3-ITD) and tyrosine kinase domain (FTL3-TKD) mutations cause constitutive activation of FLT3 and its downstream signaling pathways [2, 3]. FLT3-ITD mutations are associated with a poor prognosis, primarily due to an increased risk of relapse, with a median overall survival (OS) at five years of 30–35% [4, 5]. The clinical outcome of FLT3-ITD AML and the strong evidence of the leukemogenic role of FLT3 promoted the development of tyrosine kinase inhibitors (TKIs) [6, 7]. However, despite encouraging results achieved with targeted treatments, in association or not with chemotherapy, improvements in overall survival of FLT3-ITD^+^ AML are still modest, since most patients eventually relapse [8–11]. Acquisition of resistance to treatments involves the development of FLT3 secondary mutations [12], the evolution of leukemic clones characterized by novel mutations (especially involving the RAS pathway [13]), and protection provided by the bone marrow (BM) niche [14, 15]. These observations suggest that a possible strategy is to combine multiple approaches targeting different pro-survival pathways. Leukemic cells rely on adaptive responses to cope with intrinsic and extrinsic sources of proteotoxic stresses, that result in the disruption of protein homeostasis [16]. This is particularly important in FLT3-ITD^+^ AML cells, which are subjected to intrinsic endoplasmic reticulum (ER) stress since FLT3-ITD is a misfolded protein retained in this organelle [2]. In this context, we set up an approach based on the induction of ER and oxidative stress that showed cytotoxic activity against FLT3-ITD^+^ AML, both in cell lines and primary blasts isolated from patients [17]. This is based on the combined use of low doses of the differentiating agent retinoic acid (RA), the ER stress inducer tunicamycin (Tm), and the oxidative stress inducer arsenic trioxide (ATO) and importantly did not show toxicity on normal hematopoietic progenitors treated ex vivo. To facilitate possible translational applications, here we substituted Tm with the proteasome inhibitor bortezomib (Btz) to induce ER stress and alteration of proteostasis. RA and ATO are employed in clinical practice for acute promyelocytic leukemia (APL) [18]. APL treatment is a paradigm of targeted therapy. This subtype of AML is characterized by the fusion protein PML-RARα that impedes the differentiation transcriptional program driven by RARα. Pharmacological doses of RA are able to re-activate RARα-mediated transcription resuming differentiation and to target PML-RARα for degradation. Treatment with RA and chemotherapy is however associated with relapses, risks of severe infections and secondary leukemias [19]. RA-resistant or relapsed patients are effectively treated with a combination of RA and ATO, which mechanism of action is based on its ability to generate oxidative stress thus mediating efficient PML-RARα degradation [18, 20]. Unfortunately, although many attempts have been performed, RA can efficiently induce differentiation only in APL. However, in some AML types, RA is able to partially trigger transcriptional and translational programs that, even though not completely, activate the leukemic blast [21]. Btz is used in clinical practice in therapies for multiple myeloma (MM) and mantle cell lymphoma [22, 23]. MM is a tumor of plasma cells, terminally differentiated effectors of the B cell lineage specialized in producing large amounts of antibodies. The massive secretory apparatus necessary for normal and transformed plasma cells to allow for high-level protein secretion constitutes a distinctive therapeutic target. In fact, proteostasis imbalance is at the basis of the molecular mechanisms that render MM specifically sensitive to proteasome inhibitors, since blocking misfolded and unfolded protein degradation shifts the balance of the proteostasis pathways (like the UPR and autophagy) toward apoptosis [24, 25]. Thus, we reasoned that FLT3-ITD^+^ AML cells could be targeted in a similar manner by inducing translational activity by RA and simultaneously increasing proteotoxic stress by ATO (which causes oxidative stress) and by Btz (which impairs the protein degradation apparatus, clogging the secretory pathways with misfolded proteins). We demonstrate that the combination of RA, Btz and ATO (RBA) efficiently targets FLT3-ITD^+^ AML cells. Furthermore, since the BM niche plays a crucial role in protecting leukemic cells from the cytotoxic effects of different therapies [14, 15], we assessed the efficacy of the combination RBA on AML cells in a co-culture system with BMSCs. We found that BMSCs render AML cells totally resistant to the combination RBA, but the addition of ascorbic acid (Vitamin C) is sufficient to reduce the protective effects. We also investigated the molecular basis involved in the cross-talk between AML cells and BMSCs upon treatment demonstrating the involvement of actin cytoskeleton dynamics and of the transcriptional co-regulator YAP. Eventually, we tested the efficacy of the combination RBA plus ascorbic acid in a murine model of human FLT3-ITD^+^ AML, and found that it significantly prolongs the mice life span, reducing AML engraftment.

## Methods

A detailed Methods section is available in the supplemental material.

### Cell culture

Human AML cell lines MOLM-13, MV4-11, MOLM-14, ML-2, OCI-AML2, OCI-AML3, HL60, and NB4 were cultured in RPMI 1640 medium supplemented with 1% penicillin/streptomycin and 10% FBS. Primary murine BMSCs were obtained from the sacrifice of three healthy mice, by flushing of the femurs with a syringe. After digestion of the bone matrix by collagenase for 40 minutes, isolated cells were seeded in DMEM F12 with 20% FBS enriched with growth factors. Murine BMSC line MS-5 was cultured in MEMα with ribonucleosides and deoxyribonucleosides, with 1% penicillin/streptomycin, 20% FBS and 100 μM βmercaptoethanol. All the cultures were maintained at 37°C in a humified atmosphere containing 5% CO_2_.

### Primary cells

AML BM samples were collected at diagnosis at the Department of Biomedicine and Prevention at the University of Rome Tor Vergata, after obtaining informed consent from all patients and approval of the study by the IRB of Policlinico Tor Vergata, Rome, in accordance with the *Helsinki Declaration* of 1975, as revised in 2013. CD34^+^ LSCs from patient BM samples and CD34^+^ HSCs from healthy donors, were isolated by positive selection with CD34 MicroBead Kit UltraPure human (Miltenyi Biotec) then cultured and treated in vitro at a concentration of 10.000 cells/ml in STEM SPAN Leukemic cell expansion medium.

### Treatments

MOLM-13 cells were seeded at a concentration of 70.000 cells/ml and treated with 10nM RA, 2.25nM Btz and 500nM ATO, alone or in combination, as described in the text. RA and Btz were dissolved in DMSO, thus an equivalent amount of DMSO was added in all the samples. Primary LSC CD34^+^ and HSC CD34^+^ cells were treated with 10nM RA, 3nM Btz, and 1mM ATO. In order to attenuate oxidative stress, cells were pre-incubated for 24 h with N-Acetyl cysteine (NAC) 20mM at pH 7.4 and then treated with the above-mentioned drugs, adding again also NAC 20mM. In the co-culture system, ascorbic acid was added at a 4.5mM concentration.

### Cell death and cell proliferation

We performed flow cytometry of leukemic cell lines to assess cell death and cell proliferation by propidium iodide exclusion assay, and cell cycle by propidium iodide staining of cells fixed O/N with 70% ethanol. In the co-culture experiments, the same procedure was performed for stromal cells upon detachment with 0.05% trypsin/0.53mM EDTA.

### Evaluation of ER and oxidative stress

We performed RT-qPCR, flow cytometry, confocal microscopy, and TEM analysis [26, 27] to evaluate proteostasis alteration and the activation of the UPR at different time points. Also, we used the same techniques to assess the generation of oxidative stress. ROS levels were measured by harvesting cells and incubating them with 2μM CM-H_2_DCFDA (ThermoFisher Scientific) in pre-warmed PBS at 37°C for 30 min in the dark. The mitochondrial depolarization was measured by using JC-1 dye (ThermoFisher Scientific). The amount of the Nrf2 protein was analyzed by confocal microscopy and flow cytometry after staining fixed and permeabilized cells with an anti-Nrf2 antibody (Cell Signaling) followed by an Alexa-488 anti-rabbit antibody.

### Co-cultures

MEMα medium of MS-5 cells at 90% confluence was changed with RPMI, and 24 h later this was substituted by fresh RPMI containing MOLM-13 leukemic cells and drugs as described above. We performed flow cytometry, RT-qPCR, confocal microscopy, and WB analysis in both MOLM-13 and MS-5 cell lines to investigate the responsiveness of the co-cultures to the treatments at different time points. We then compared these results with the ones obtained in BMSC treated alone. YAP down-regulation in MS-5 stromal cells was obtained by transfecting the cells with lipofectamine (RNAiMAX) and 5nM f.c. siRNA targeting YAP or with 5nM f.c. negative control siRNA.

### In vivo mouse studies

Orthotopic human leukemia was induced by injecting 500.000 MOLM-13 cells in the tail vein of NSG mice. Treatments started 2 days after engraftment and mice were monitored for weight and behavior. After sacrifice, organs were collected for histological analysis and the bone marrow was flushed from the femurs and stained with a FITC-anti-hCD45 antibody. The study was approved by the Italian Ministry of Health.

## Results

### The combination of low doses of Retinoic Acid, Bortezomib and Arsenic Trioxide induces cell death of FLT3-ITD^+^ AML cell lines and primary human leukemic stem cells

**Fig. 1:**
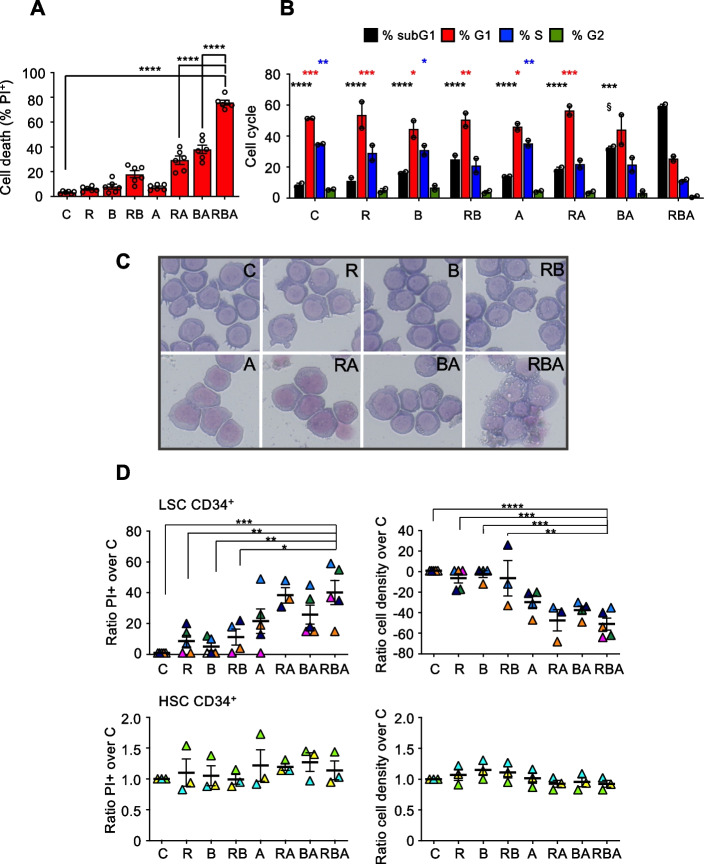
The FLT3-ITD^+^ MOLM-13 cells and primary leukemic stem cells are sensitive to the combination of RA, Btz and ATO. **A** MOLM-13 AML cells were treated for 72 h with 10nM RA, 2.25nM Btz, and 500nM ATO, alone or in combination. In all the figures retinoic acid is indicated as R, bortezomib as B, and ATO as A. Cell death was assessed by propidium iodide (PI) exclusion assay, analyzed by flow cytometry (one-way ANOVA). **B** Cell cycle analysis of MOLM-13 cells 48 h after treatments as in (A). The cells treated with BA show an increased percentage of subG1 phase relative to C cells but only those treated with RBA exhibit significant modulation of all the cell cycle phases. (the asterisks refer to two-way ANOVA analysis of each sample compared vs RBA, and colors are in accordance with the corresponding phase of the cell cycle; § indicates two-way ANOVA analysis of subG1 BA vs subG1 C: *P* < 0.005). **C** MOLM-13 cell morphology analyzed by Wright-Giemsa staining, 72 h after treatments. **D** FLT3-ITD^+^ CD34^+^ cells, isolated from patients at diagnosis (*n* = 5 upper panels) or from healthy volunteers (*n* = 3 lower panels), were treated ex vivo for 96 h with 10nM RA, 3nM Btz, and 500nM ATO, alone or in combination. Cell death (left) and cell density (right) were measured by flow cytometry after staining with PI (one-way ANOVA)

We screened a panel of human AML cell lines to assess their sensitivity to the combination of RA, Btz, and ATO (RBA). As we observed in our previous work [17], the cell lines expressing FLT3-ITD resulted to be the most affected by the induction of proteotoxic stress (Supplemental Fig. 1). We chose doses of each drug that did not cause significant cell death or cell cycle alterations when used alone, but only in combination. The triple combination exerted cytotoxic effects on the FLT3-ITD^+^ MOLM-13 (Figs. 1A, B, and C), MOLM-14, and MV-4–11 cell lines (Supplemental Fig. 1). We then tested the combination on FLT3-ITD^+^ primary AML leukemic stem cells (CD34^+^) and found that these are equally sensitive to the double and triple combinations of the drugs (Fig. 1D). Importantly, the efficacy of the drugs is specific for leukemic stem cells, since the same treatments did not alter CD34^+^ healthy hematopoietic stem cells’ viability and proliferation rate (Fig. 1D).

### Treatment with the combination RBA alters ER homeostasis in MOLM-13 cells but the adaptive branch of the UPR is suppressed

**Fig. 2:**
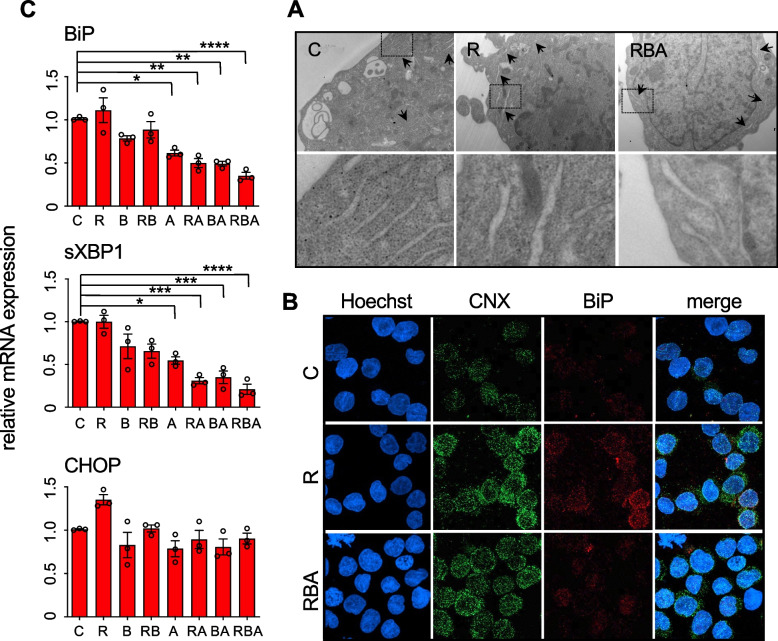
RBA combined treatment generates ER stress without consequent activation of the pro-survival UPR pathway. **A** TEM analysis of MOLM-13 cells, 24 h after treatment, shows swollen ER in the cells treated with R alone and with the combination RBA relative to C cells. Black arrows point to ER tubules. Dashed squares in the upper panels indicate the areas magnified in the lower panels. **B** Confocal microscopy images of MOLM-13 cells, 24 h after treatment, stained with anti-calnexin (CNX) and anti-BiP antibodies. **C** Expression levels of BiP, sXBP1, and CHOP genes, 48 h after treatment, measured by RT-qPCR (one-way ANOVA)

Since the triple combination exerted stronger effects on MOLM-13 than on MOLM-14 cells, and the MV-4–11 showed some sensitivity to retinoic acid alone, we chose the MOLM-13 cell line as a model system for the following analysis. We expected that the combination RBA would cause proteotoxic stress because the three drugs affect ER functions and protein folding. Although AML subtypes like MOLM-13 cells do not respond to the differentiation stimulus provided by retinoic acid in such a complete manner as acute promyelocytic leukemia cells do [28], it could still induce partial differentiation by increasing ER activity. Indeed, we observed signs of differentiation and activation of the secretory pathway upon retinoic acid administration, as indicated by diminished nucleus/cytosol ratio and cytosolic basophilia (Fig. 1C), swollen ER tubules (Fig. 2A), and augmented expression of the ER chaperones calnexin and BiP (Fig. 2B). Since Btz inhibits the 26S subunit of proteasome, it blocks the final step of the ER-associated degradation (ERAD), the process through which misfolded proteins accumulated in the ER are eventually degraded [29]. Thus, by administrating Btz we expected to induce proteostasis imbalance, affecting ER protein folding capacity. Furthermore, ATO induces oxidative stress, which impairs protein folding [30]. Indeed, MOLM-13 cells treated with RBA present swollen ER tubules (Fig. 2A) and increased expression of the ER chaperone calnexin (Fig. 2B), indications of increased activity of the ER. ER stress is defined as the accumulation of misfolded/unfolded proteins in the ER lumen exceeding the folding capacity of the ER. Such circumstances trigger a series of signaling pathways, collectively known as the unfolded protein response (UPR), which regulate the expression of genes involved in protein folding and secretion to re-establish ER homeostasis. However, in case of prolonged or overwhelming stress, the UPR leads to apoptotic cell death [31]. Surprisingly, despite increased ER activity, MOLM-13 cells treated with RBA not only do not up-regulate the expression of the two main UPR players involved in homeostasis recovery, namely the ER chaperone BiP and the spliced form of the transcription factor XBP1, but this is down-regulated, further worsening ER proteostasis imbalance, whereas the pro-apoptotic UPR gene CHOP is not modulated (Fig. 2C and Supplemental Fig. 2). The same down-regulation of BiP and sXBP1 is observed upon administration of ATO, alone or in combination with RA or Btz, indicating induction of proteotoxic stress.

### Treatment with the combination RBA induces oxidative stress, which is the main driver of its cytotoxic effects

**Fig. 3:**
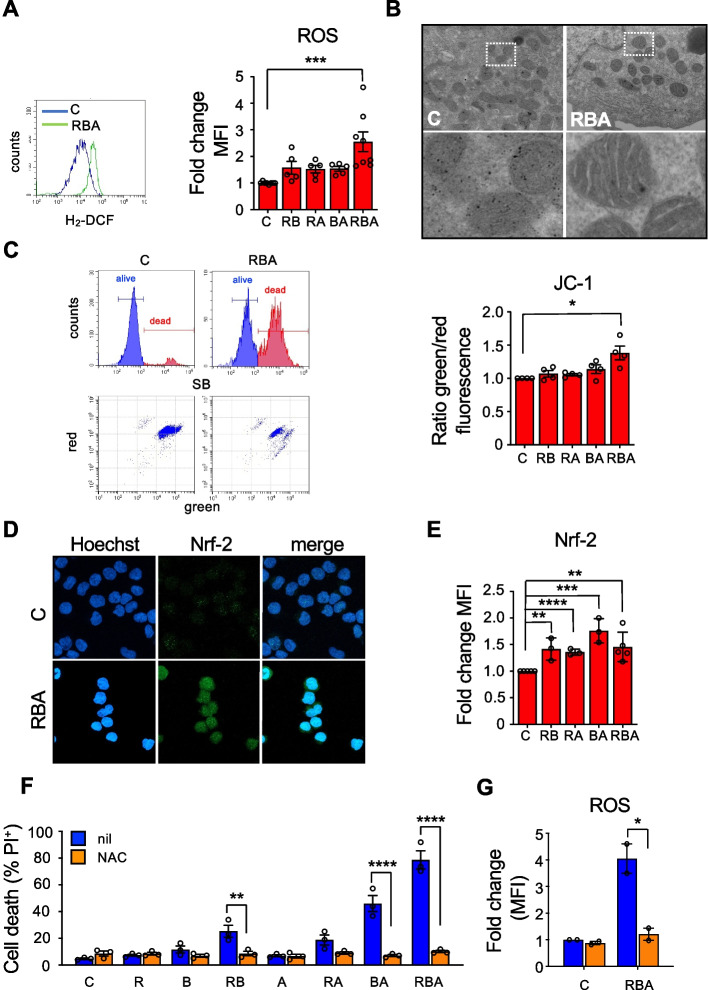
Oxidative stress is mostly responsible for the cytotoxic effect of the combination on MOLM-13 cells. **A** ROS levels were quantified in MOLM-13 cells by flow cytometry, upon the incorporation of the oxidation-sensitive dye CMH_2_-DCFDA, 48 h after RBA treatment (one-way ANOVA). **B** TEM analysis focused on mitochondria of MOLM-13 cells, 24 h after treatment; areas delimited in dashed squares in the upper panels are magnified in the lower panels. **C** MOLM-13 cells mitochondrial membrane depolarization, measured by flow cytometry as increased green/red fluorescence ratio of the JC-1 dye, 48 h after treatments. On the left are shown representative flow cytometry plots, on the right the histogram reports average values of the green/red MFI ratio. **D** Confocal microscopy images of MOLM-13 cells stained with an anti-NRF-2 antibody. **E** NRF-2 expression in MOLM-13 cells, 24h after treatment, measured by flow cytometry (unpaired Student’s T test). **F** MOLM-13 cell death was measured by PI exclusion assay, 72 h after treatment with RBA, in the absence or in the presence of 20mM N-acetyl-cysteine (left, two-way ANOVA). **G** ROS measurement, in cells treated with or without NAC, for 48 h (right, two-way ANOVA)

ATO causes oxidative stress and ER and oxidative stress induce one another, although the underlying mechanisms are not completely clear yet [30, 32]. Hence, we measured the levels of reactive oxygen species (ROS) in MOLM-13 cells treated with the double and triple combinations and found that only RBA caused their significant increase (Fig. 3A). Oxidative stress damages mitochondria by affecting the mitochondrial respiratory chain, altering membrane permeability and calcium homeostasis, and weakening the mitochondrial defense system [33]. Accordingly, the combination RBA causes alterations in mitochondria, as shown by disrupted cristae morphology observed by TEM, a sign of mitochondria suffering (Fig. 3B), and by depolarization of the mitochondria membrane, as measured by the JC1 dye (Fig. 3C). In the presence of oxidative stress, cells trigger the oxidative stress response, mainly orchestrated by the transcription factor Nrf-2. All the double and triple combinations up-regulate the expression of Nrf2 (Fig. 3D, E, and Supplemental Fig. 2). These data together suggest that the triple and the double combinations induce oxidative stress and elicit an oxidative stress response, that however is not enough to reduce oxidative stress only upon treatment with the triple combination. These data indicate that proteotoxic stress resulting from RA-mediated ER activation and the hindrance of ERAD by Btz generates oxidative stress, that is further worsened by the addition of ATO. Very importantly, the reduction of oxidative stress by the addition of the reducing agent N-acetylcysteine is sufficient to render MOLM-13 cells resistant to all the combinations of RA, Btz, and ATO, including the triple one (Fig. 3F, G, and Supplemental Fig. 2). This is not surprising since we have already demonstrated that the reduction of oxidative stress allows AML cells to better cope with proteotoxic stress and survive [17]. Furthermore, a pivotal work by Han and colleagues shows that the main driver of cell death upon ER stress is the generation of oxidative stress [34].

### Murine bone marrow stromal cells protect MOLM-13 cells from the toxic effects of the combination RBA, but ascorbic acid overcomes this defense

**Fig. 4:**
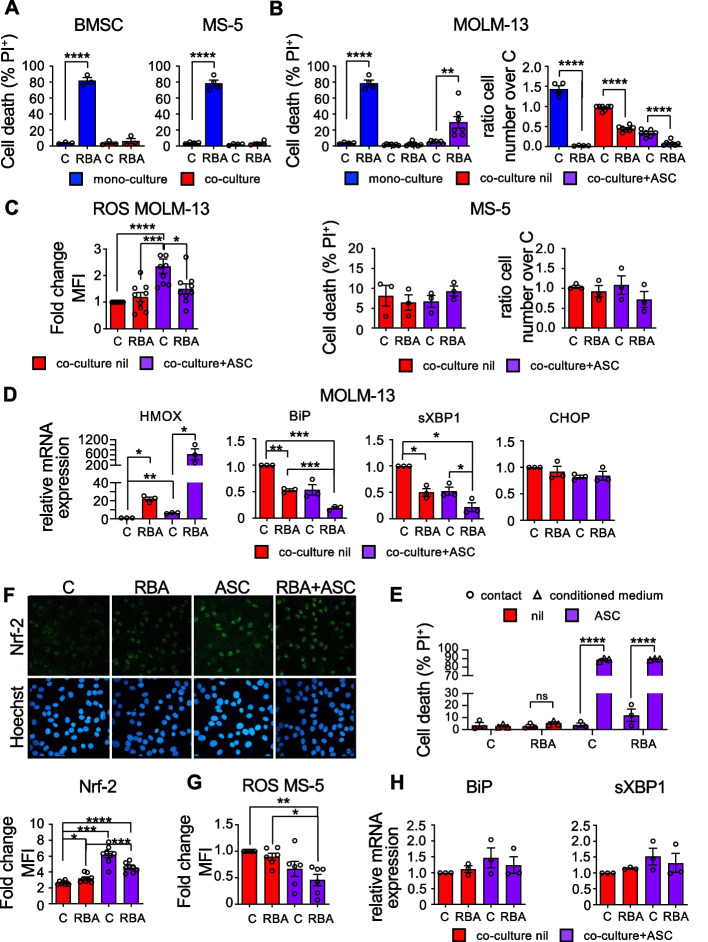
Murine bone marrow stromal cells nullify the effect of the combination RBA on MOLM-13 cells, but ascorbic acid restores it. **A** PI exclusion assay showing cell death of MOLM-13 leukemic cells treated with 10nM RA, 2.25nM Btz, and 500nm ATO (RBA) for 72 h, in mono-culture (blue) or co-culture (red) with primary BMSCs and MS-5 stromal cell line, (left and right panel respectively, one-way ANOVA). **B** Cell death (left) and cell proliferation (right) of MOLM-13 cells (upper panels) and MS-5 cells (lower panels) treated in co-culture with RBA and 4.5mM ascorbic acid (ASC), for 72 h (one-way ANOVA). MOLM-13 cells, treated in mono-culture, are shown as control (blue bars). **C** MOLM-13 cells were treated in co-culture with MS-5 cells as in (B), and ROS levels were measured after 72 h. **D** HMOX, BiP, sXBP1, and CHOP mRNA expression levels of MOLM-13 cells, treated for 24 h as in (B) (one-way ANOVA). **E** MOLM-13 and MS-5 cells were treated with the combination RBA and ASC as in (B) in co-culture. After 24 h, the conditioned medium (CM) was collected and used to treat MOLM-13 cells in mono-culture. The graph shows cell death of MOLM-13 cells treated for 48 h with CM (one-way ANOVA). **F** MS-5 cells were treated in co-culture with MOLM-13 for 72 h, then analyzed for Nrf-2 expression by confocal microscopy. The histogram reports the quantification of Nrf-2 mean fluorescence intensity (n = 8 fields ± S.E.M., one-way ANOVA). **G** ROS measurement in MS-5 cells from the co-culture by flow cytometry (one-way ANOVA). **H** Assessment of BiP and sXBP1 mRNA expression levels in MS-5 cells from the co-culture, 24 h after treatment (one-way ANOVA)

The BM niche is a heterogenous microenvironment in which normal HSCs reside and communicate with other cell populations through both direct contact and paracrine signals [35]. This crosstalk is essential for determining HSC fate and maintaining the balance between self-renewal and differentiation. Likewise, malignant LSCs interact with the cells populating the niche, altering the composition of the microenvironment to deceive the immune system, proliferate indefinitely, and invade the BM and other organs. BMSCs play a main role in supporting AML progression and resistance to therapies by a variety of mechanisms [14], most still to be elucidated, among which enhancing AML cells’ antioxidant defenses [15]. Thus, it was essential to assess the efficacy of the combination RBA on AML cells in their presence. To this aim, we co-cultured MOLM-13 cells with mouse primary BM mesenchymal stem cells/BMSCs freshly isolated or with the mouse BM stromal cell line MS-5. We chose murine cells in the perspective to use the combination in murine models of human leukemia (see below). In both cases, the stromal cells completely protected MOLM-13 cells from the effects of the combination RBA (Fig. 4A). Since oxidative stress plays a pivotal role in the toxicity of the combination RBA, and based on literature we expected that BMSCs would enhance AML cells’ antioxidant defenses, we looked for an adjuvant to further increase oxidative stress, without causing general toxicity. We chose to use ascorbic acid, or vitamin C. Ascorbic acid (ASC) is known as an anti-oxidant, which is true at micromolar concentrations. However, different studies show that at millimolar concentrations it causes oxidative stress and it has been used in clinical trials as an adjuvant of chemotherapy, without generating toxic effects [36, 37]. Addition of ASC to the combination RBA completely rescued its cytostatic effects and partially its cytotoxic effects on MOLM-13 cells (Fig. 4B upper panels) without affecting the viability or proliferation of MS-5 stromal cells (Fig. 4B lower panels). ROS levels resulted low in MOLM-13 cells 72h after treatment with RBA or RBA plus ASC, showing just a slight increase in the cells treated with ASC alone (Fig. 4C). However, the extensive increase of HMOX expression, a main target gene of the anti-oxidant stress response driven by Nrf-2, indicates the presence of high levels of oxidative stress in MOLM-13 treated with the combination RBA plus ASC (Fig. 4D). A very strong antioxidant response could explain the low amounts of ROS. On the contrary, HMOX is only slightly up-regulated in MOLM-13 treated with ASC alone. Treatment with the combination RBA or with ASC alone partially affects MOLM-13 ER homeostasis, as indicated by the reduction of the expression of BiP and sXBP-1 (Fig. 4D). However, their down-regulation is much stronger in cells treated with the combination RBA plus ASC. Altogether these data indicate that MS-5 stromal cells protect MOLM-13 from the combination RBA or ASC alone reducing the levels of oxidative stress and preserving the functions of the ER, so the cells are only partially affected by the treatments and just slow down proliferation (Fig. 4B). In this context, it is important to note that the concentration of ASC we used is dramatically toxic for MOLM-13 cells in mono-culture (data not shown). Addition of ASC to the combination RBA repristinates amounts of oxidative stress high enough that cannot be recovered by stromal cells and that worsen proteotoxic stress in MOLM-13 cells. Consequently, the latter undergo cell death and arrest of proliferation. Importantly, we observed that medium conditioned by a co-culture of MS-5 with MOLM-13 is sufficient to defend MOLM-13 cells by the combination RBA in mono-culture, but cell–cell contact is necessary to protect from ASC and RBA plus ASC (Fig. 4E).

### Mouse stromal cells treated in co-culture with MOLM-13 cells activate the oxidative stress response, undergo disruption of the actin cytoskeleton, and relocalize YAP in the cytosol

**Fig. 5:**
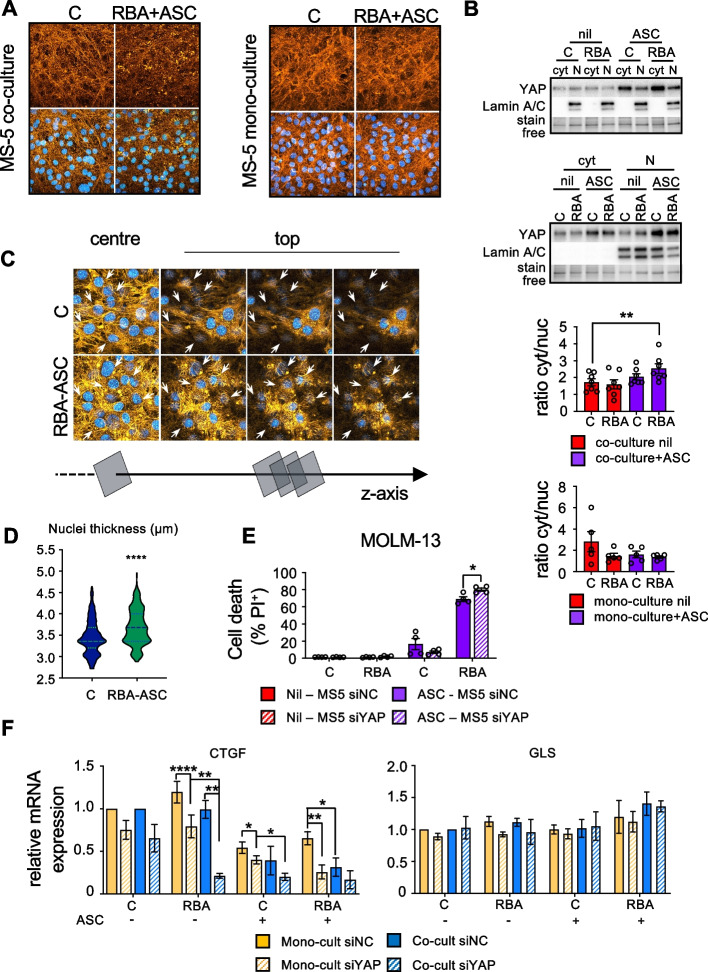
BMSCs undergo cytoskeletal rearrangements and re-localize YAP in the cytosol when treated with the combination RBA plus ASC in co-culture with leukemic cells. **A** The right panels show representative images of MS-5 cells treated in co-culture with MOLM-13 for 72 h, then stained with phalloidin to detect F-actin (orange). Cells were counterstained with Hoechst to identify nuclei (blue). The right panels show the same analysis, but MS-5 cells were treated with the combination RBA plus ASC in the absence of the MOLM-13 cells. **B** YAP protein levels in MS-5 cells, treated in co-culture with MOLM-13 cells (upper panel) or in mono-culture (lower panel), were evaluated by western blot analysis, upon nucleus-cytosol fractionation. Lamin A/C was used as nuclear marker and stain free protein detection technology (BioRad) for total protein normalization. The graphs report the average ratio of YAP amount in the cytosol over that in the nucleus (paired Student’s T test). **C** Single confocal images of a Z stack, taken in the center or in the apical portion of MS-5 cells, stained as in (A), to examine the actin cap. **D** The violin plot reports the measurements of MS-5 nuclear thickness, obtained by the confocal analysis shown in (C) and in Supplemental Fig. 6. **E** PI exclusion assay showing cell death of MOLM-13 leukemic cells co-cultured for 72 h with MS-5 cells silenced (siYAP) or not (siNC) for YAP protein (two-way ANOVA). **F** CTGF and GLS mRNA expression levels of MS-5 cells as mono- or co-culture with MOLM-13 cells, upon 72 h of treatment (two-way ANOVA)

To investigate the mechanisms underlying the protection provided to AML cells, we assessed the effects of the treatments on MS-5 cells in co-culture with MOLM-13 cells. Interestingly, we observed that MS-5 cells activate their anti-oxidant defenses as indicated by up-regulation of Nrf-2, slightly upon treatment with RBA alone and more with ASC or RBA plus ASC (Fig. 4F); ROS levels decrease accordingly (Fig. 4G). The UPR is not perturbed by the treatments in MS-5 since we did not detect changes in BiP or sXBP-1 expression, confirming that MS-5 cells are not as affected as MOLM-13 (Fig. 4H). Although, as shown above, we found no differences in the viability or proliferation of MS-5 cells (Fig. 4B), we noticed modifications in their morphology (data not shown). This prompted us to analyze the structure of the actin cytoskeleton and surprisingly we found that the treatment with RBA plus ASC in co-culture caused significant disruption, with evident shortening of actin microfilaments (Fig. 5A left panel and Supplemental Fig. 5). As mentioned above, leukemic cells alter BMSC functions to their advantage, thus we wondered if actin cytoskeleton modifications were simply due to the treatments or because of the crosstalk with MOLM-13 in response to the treatments. Importantly, MS-5 cells cultured and treated alone do not show any change in their actin cytoskeleton, suggesting that this phenomenon is a consequence of the interaction with MOLM-13 in response to stress (Fig. 5A right panel).

It is well assessed that transformations in the mechanical environment and actin cytoskeleton regulate the transcriptional activator YAP through Hippo pathway dependent and independent mechanisms [38]. In particular, when the actin cytoskeleton is well developed, with an abundance of actin microfilaments and stress fibers, YAP is localized in the nucleus and is transcriptionally active. On the contrary, when the actin cytoskeleton is disrupted (for example when cells grow in a soft extracellular matrix (ECM) or onto small surfaces or at high density) YAP is relocalized in the cytosol and its target genes are silenced [39, 40]. Thus, we measured the ratio between cytosolic and nuclear YAP in MS-5 cells upon treatment in co-culture with MOLM-13. We found that YAP is partially relocalized in the cytosol of cells treated with RBA plus ASC, indicating downregulation of its activity (Fig. 5B upper panel). Accordingly to our observations relative to the actin cytoskeleton, MS-5 cells treated alone do not relocalize YAP in the cytosol, meaning that cytoskeletal disruption and YAP modulation are due to the interaction with MOLM-13 cells (Fig. 5B lower panel). The regulation of YAP localization and activity in relation to the actin cytoskeleton is quite complex, regulated at multiple levels, and not completely elucidated yet [38, 41, 42]. Recent works show that YAP transcriptional activity and actin structure could be involved in a feedback loop regulating the integrity of the nuclear envelope [43]. Moreover, it has been shown that a flatter shape of the nucleus, which is maintained by a well-developed actin cap [44], favors YAP entry into the nucleus [45, 46]. Thus, we analyzed by confocal microscopy the structure of the nuclear actin cap of MS-5 cells treated with RBA plus ASC in the presence of MOLM-13 cells and found that it is disrupted, characterized by short and disordered actin filaments, contrary to untreated cells in which the actin filaments on top of the nuclei are elongated and orderly integrated into the actin network (Fig. 5C). As a consequence, the nuclei thickness of treated cells is significantly augmented. The graph shows the expansion of subpopulations of MS-5 cells presenting nuclei with heightened thickness and a decrease in those with shorter one (Fig. 5D and Supplemental Fig. 6). In order to assess if the translocation of YAP into the cytosol was correlated with lower protection provided by the MS-5 to the MOLM-13 cells, we down-regulated YAP expression in MS-5 cells (Supplemental Fig. 7) and evaluated the response to the treatment of MOLM-13 in co-culture with MS-5 transfected with a negative control siRNA (siNC) or with a siRNA targeting YAP (siYAP) (Fig. 5E). We noticed increased rates of death of MOLM-13 treated with RBA plus ASC in co-culture with both siNC or siYAP MS-5, likely due to the fact that transfection per se causes stress, rendering the MS-5 cells weaker in protecting the MOLM-13 cells. Importantly, the down-regulation of YAP in MS-5 further tampered with their ability to defend MOLM-13 cells by the treatment. Eventually, in order to confirm that the partial translocation of YAP observed in MS-5 cells upon treatment in co-culture would affect the expression of YAP target genes, we evaluated the expression of a well-established one, CTGF [39, 47]. The data confirm that CTGF is a YAP target gene in the MS-5 cells since in siYAP MS-5 its expression is decreased (Fig. 5F left panel). According to the translocation of YAP from the nucleus, CTGF expression is lower in MS-5 treated in co-culture with MOLM-13 cells than in those treated in the absence of MOLM-13 cells. As a control, we show that GLS, which is a YAP target in other cell types [39] but not in MS-5, as indicated by the fact that it is not modulated in siYAP MS-5, is expressed at the same levels in MS-5 upon treatment with RBA plus ASC in the presence or in the absence of MOLM-13.

**Fig. 6:**
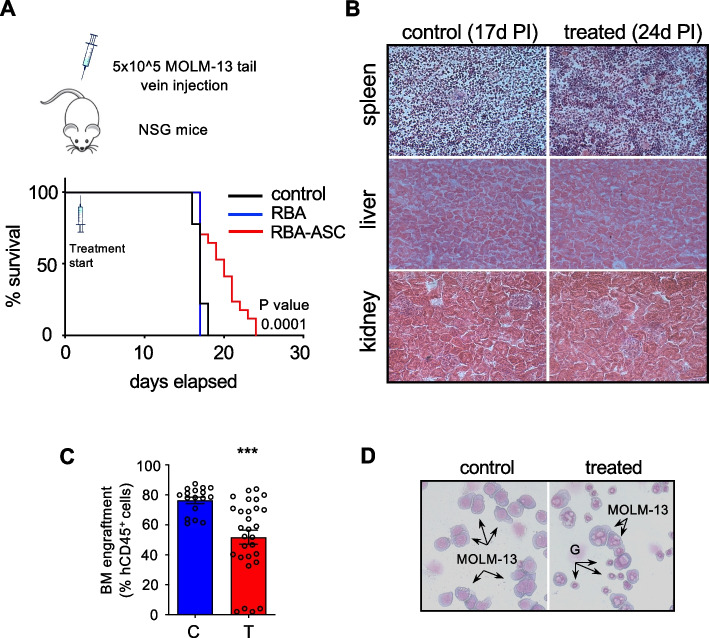
The combination of RBA plus ASC acid significantly prolongs the life span of NSG mice engrafted with human FLT3-ITD^+^ MOLM-13 cells. **A** Kaplan-Meyer survival plot showing OS of NSG mice engrafted with MOLM-13 leukemic cell line and treated with vehicle, RBA, or RBA + ASC, starting at day 2 after cell injection. (control = 12, RBA = 5 and RBA + ASC = 17 from three independent experiments). **B** Histological analysis of the spleen, liver, and kidney of NSG engrafted mice, treated or not with the combination RBA plus ASC, show no signs of toxicity due to the treatment. **C** The treatment with RBA plus ASC reduces the percentage of leukemic cells in the BM of engrafted mice. Both control and treated mice were sacrificed when showing posterior limb paralysis. Bone marrow was flushed by femurs and purified mononucleated cells were analyzed by flow cytometry after staining with an anti-human CD45 antibody (unpaired Student’s T test). **D** Wright-Giemsa staining of the cells collected from mice bone marrow shows extensive vacuolation of MOLM-13 cells isolated from the BM of treated mice present to a much lesser extent in those obtained from control ones. G indicates murine granulocytes that show no vacuolation

Altogether our data suggest that the crosstalk between AML cells and stromal cells, in the presence of stress generated by the combination RBA plus ASC, induces changes in the stromal cells that activate the oxidative stress response and undergo disruption of the actin cytoskeleton. As a consequence, part of the stromal cells presents rounder nuclei which could be one of the mechanisms underlying partial YAP inactivation by cytosolic relocalization.

### The combination RBA plus ascorbic acid prolongs the life span of a murine model of human AML

To assess the efficacy, and possible toxicity, of the combination RBA plus ASC in a murine orthotopic model of human AML in vivo, we exploited the NSG mouse model engrafted with MOLM-13 cells. We observed a significant increase in the life span of mice treated with RBA plus ASC (Fig. 6A) compared to engrafted, untreated animals, without differences in behavior, body weight, and tissue morphology of the spleen, liver, and kidney (Fig. 6B). According to our observations in co-culture conditions, treatment with the combination RBA alone had no effects (Fig. 6A). Importantly, analysis of the femurs’ bone marrow at the time of sacrifice evidenced significantly lower leukemia engraftment in treated mice (Fig. 6C and Supplemental Fig. 7B). Furthermore, most of the MOLM-13 cells recovered from the treated mice show extensive vacuolation (but the murine granulocytes do not), contrary to the cells recovered from the control mice, in which just a small minority of MOLM-13 cells present such vacuoles. These findings indicate that the treatment RBA plus ascorbic acid specifically hits AML cells in the BM in vivo, without evident general toxicity.

## Discussion

The frontline of AML cure is focusing on molecular targeted therapy although, at present, the development of resistance and consequent relapses remain major issues [48]. Clonal selection is one of the main obstacles to a successful cure [13]. The most promising strategy to restrict this phenomenon is to target different oncogenic/survival pathways in combination. Protein homeostasis is essential for cell survival and leukemia cells depend on adaptive stress responses [16, 49, 50]. We have already demonstrated that FLT3-ITD^+^ AML cells are particularly sensitive to proteostasis imbalance, also because this mutated protein is misfolded and retained in the ER by its quality control systems [17]. To increase the translational potential of our strategy we use here the proteasome inhibitor bortezomib to induce proteotoxic stress, in combination with retinoic acid and arsenic trioxide, since, as the last two, Btz is already available in clinical practice. Using a combination of drugs at low doses targeting different pathways, all inducing proteotoxic stress, should increase specificity and decrease off-target toxicity. This is supported by our observation showing, both in vitro and in vivo*,* strong effects on FLT3-ITD^+^ AML cells but not on other cell types or organs. Indeed healthy CD34^+^ HSCs (Fig. 1D lower panels) and the murine BMSC line MS-5 (Fig. 4B, lower panels) present no alterations of cell viability and proliferation upon treatment, and histological analysis of spleen, liver, and kidney of NSG mice engrafted with MOLM13 cells, treated or not with the combination RBA plus ASC, displays no differences. It is to be noted that whereas we observe consistently higher efficacy of the triple combination RBA, relative to the double ones RB, RA, and BA in different FLT-ITD^+^ cell lines (Supplemental Fig. 1), the combinations RA, BA, and RBA, equally affect primary CD34^+^ LSCs. We attribute their stronger effects on primary cells to their lack of adaptation to the higher oxygen percentage present in culture conditions than that in the bone marrow, since the main driver of cytotoxicity of these treatments is the generation of oxidative stress. Further investigation in pdx murine models is needed to clarify if the triple combination is more efficacious than the double ones also on LSCs as it is on cell lines.

In addition to clonal selection, the complex framework of the disease comprises crosstalk between AML cells and the BM niche upon therapy administration [14]. In fact, we observed that BMSC protect AML cells from the cytotoxic effects of the combined treatment. Our findings, indicating that oxidative stress is at the basis of the efficacy of the combination RBA, suggested using ascorbic acid as an adjuvant to exacerbate it and overcome resistance. In this context, it is clear the importance of understanding the mechanisms underlying AML-stromal cell crosstalk. AML cells and BMSCs exchange cytosolic content by tunneling nanotubes (TNT), which are formed by AML cells and take contact with BMSCs [51]. These are sustained by an actin structure [52], mediate the transfer of mitochondria from BMSCs to AML cells and increase the latter anti-oxidant defenses and resistance to chemotherapy [15, 51]. Our study reveals that the combination RBA plus ascorbic acid disrupts the structure of the BMSC actin cytoskeleton. Although the molecular mechanism must still be investigated, this could play a main role in the ability of this treatment to overcome the resistance of AML cells provided by the BMSC. It is important to underline that actin cytoskeleton disruption in MS-5 is not merely the effect of the stress generated by the combination RBA plus ASC, but it is a consequence of the interaction between MOLM-13 and MS-5 cells in condition of stress. Notably, actin cytoskeleton structure is closely linked to the activity of the transcriptional co-regulator YAP, which plays a main role in development, tissue homeostasis, and cancer. We observe that, upon treatment of the co-cultures, MS-5 cells partially relocalize YAP in the cytosol, thus restricting its activity. Moreover, the down-regulation of YAP in MS-5 cells weakens their protective effects on AML. To our knowledge, this is the first time in which YAP is found to be involved in the crosstalk between AML and BM stromal cells.

Many critical questions remain to be answered and are already matters of investigation in our lab. The first concerns the meaning of YAP relocalization in MS-5 upon treatment with RBA plus ASC. It will be of paramount importance to understand the role of YAP in the leukemic niche since manipulation of its activity in the BM environment could become an important therapeutic strategy. The second relevant issue to be investigated regards the mechanisms determining actin cytoskeleton disruption. It has been shown that AML cells promote mitochondrial transfer from BMSCs, via TNT, by increasing their levels of oxidative stress via NOX2 activity [51]. The combination RBA plus ASC dramatically alters the redox balance of AML cells and this could have consequences on actin remodeling in BMSCs, which is involved in TNT formation. Another possibility could be that the interaction of stromal cells with leukemic cells undergoing stress causes ECM rearrangements, rendering it “softer”, which would induce actin cytoskeleton disruption and YAP cytosolic relocalization in stromal cells [38, 42]. The BM ECM is modified in AML and myelodysplastic syndrome [53, 54], and AML cells produce a different secretome than HSCs or normal progenitors [54, 55]. The combination RBA plus ASC could interfere with this equilibrium that provides advantages to AML cells, with consequences on stromal cell mechanotransduction. The last and more important concern to be further investigated regards the efficacy of the treatments in pdx mouse models.

## Conclusions

On the whole, we report the possibility of specifically targeting FLT3-ITD^+^ AML cells by inducing proteotoxic stress with the combination RBA plus ascorbic acid, all drugs already in use in clinical practice in different settings. Furthermore, we show that the inability of BMSCs to protect AML cells from the combination correlates with actin cytoskeleton disruption and YAP relocalization into the cytosol, suggesting for the first time that this mechanism could be involved in the protective functions of the BMSCs and opening to the identification of novel therapeutic targets.

### Supplementary Information

**Additional file 1.**

## Data Availability

All data supporting the findings of this study are available within the paper and its Supplementary Information.

## Abbreviations

AML: Acute myeloid leukemia
APL: Acute promyelocytic leukemia
ITD: Internal tandem duplication
ER: Endoplasmic reticulum
UPR: Unfolded protein response
RA: Retinoic acid
Btz: Bortezomib
ATO: Arsenic trioxide
RBA: Retinoic acid-bortezomib-arsenic trioxide
BMSCs: Bone marrow stromal cells
Tm: Tunicamycin
MM: Multiple myeloma
ROS: Reactive oxygen species
HSC: Hematopoietic stem cells
LSC: Leukemic stem cells
ASC: Ascorbic acid
